# Gender equality in sickness absence tolerance: Attitudes and norms of sickness absence are not different for men and women

**DOI:** 10.1371/journal.pone.0200788

**Published:** 2018-08-01

**Authors:** Gøril Kvamme Løset, Harald Dale-Olsen, Tale Hellevik, Arne Mastekaasa, Tilmann von Soest, Kjersti Misje Østbakken

**Affiliations:** 1 Norwegian Social Research (NOVA), Centre for Welfare and Labour Research, OsloMet–Oslo Metropolitan University, Oslo, Norway; 2 Institute for Social Research, Oslo, Norway; 3 Department of Sociology and Human Geography, Faculty of Social Sciences, University of Oslo, Oslo, Norway; 4 Department of Psychology, Faculty of Social Sciences, University of Oslo, Oslo, Norway; Brown University, UNITED STATES

## Abstract

Previous research offers limited understanding as to why sickness absence is higher among women than among men, but attitudes and norms have been suggested as plausible explanations of this gender gap. The purpose of the present study is to examine whether the gender gap in sickness absence reflects gender differences in sickness absence attitudes or gendered norms of sickness absence in society. The analyses are based on data from a factorial survey experiment covering 1,800 male and female employed respondents in Norway in 2016. Each participant was asked to evaluate whether sick leave would be reasonable in six unique, hypothetical sickness absence scenarios (i.e. vignettes) in which occupation, gender and reason for sick leave varied. Sick leave judgments were regressed on respondent gender and vignette gender using binary logistic regressions across three cut points. Overall, we did not find a substantial gender difference in either attitudes towards sickness absence or sickness absence norms. However, further analyses indicated more tolerant social norms of sickness absence for employees in gender-dominated occupations than for employees in gender-integrated occupations. This pattern could be a result of the type of work attributed to these occupations rather than their gender composition. Contrary to popular belief, we conclude that widely held attitudes and norms of sickness absence are unlikely to be drivers of the gender gap in sickness absence. The results can be useful for policies and interventions aimed at safeguarding gender equality in the labour market.

## Introduction

Research has repeatedly shown substantial gender gaps in sickness absence from work. For example, findings from a study examining 17 European countries showed higher sickness absence among women in all countries. Women had, on average, more than a 30% higher probability of being absent from work because of health complaints in any given week than men [1]. Similar differences are found in the US [2] and Canada [3]. Hence, the difference in sickness absence between men and women exists across different political regimes, social security systems and sick-pay policies [1,4]. Despite decades of research attempting to explain this gender difference, the phenomenon is not fully understood [5,6]. Knowledge about reasons for the higher prevalence of absence among women than men is important, as sickness absence is considered a substantial expense in Western economies [7]. Moreover, the gender gap in sickness absence could also constitute a barrier for women in the labour market [8].

Past studies on gender differences in sickness absence have mainly focused on factors that may cause women to have more health problems or be more susceptible to illness than men, and health issues related to pregnancy do indeed seem to account for part of the gender gap [8,9]. However, other health-related explanations have received limited empirical support, with neither heavier work/family loads among women than among men [10,11] nor differing work conditions for women and men appearing to be of major importance for the gender difference [12,13]. Thus, the gender gap in sickness absence remains largely unexplained [2,6].

The limited understanding of the gender difference in sickness absence warrants closer examination of motivational and attitudinal factors, which have so far received less attention as an explanation for this gender difference. A medical condition could make it impossible to attend work, yet, more typically, the individual has some degree of choice [14]. Studies show that tolerant attitudes towards work absence are actually related to higher likelihood of absenteeism the previous year [15] and number of absence days from work the previous six months [16,17]. Sickness absence without certification from a physician (self-certified sickness absence) is considered more sensitive to individual motivation, and less determined by health status, than is physician-certified sickness absence [18]. Still, even physician-certified sickness absence seems to be in part a matter of subjective decision-making, both by the patient and by the physician [19,20]. A Norwegian study also shows that in the large majority of cases, if a patient asks for sick leave, the physician will grant it [21].

Although the role of attitudinal factors in sickness absence behaviour is quite well established, such factors may not be relevant for explaining specifically the gender differences in this behaviour. However, higher sickness absence among women than among men would be expected if one of the following conditions also holds; (1) that women have more tolerant attitudes toward sickness absence than men, or (2) that the general attitudes (or social norms) in the population, which both men and women face, are more accepting with regard to women’s sickness absence. Very few empirical studies have addressed this topic [2,6]. In the present study we use Norwegian data from a large-scale factorial survey experiment in order to examine (a) how women and men judge sickness absence in different contexts; (b) whether women and men are judged differently when absent because of sickness in different contexts; and (c) whether working in female- versus male-dominated occupations influences judgments of sickness absence legitimacy.

The gender difference in sickness absence is similar to gender differences in other illness behaviours, such as help-seeking and use of medical services [22–24]. A better understanding of the role of attitudes and norms in connection with sickness absence may thus also contribute to our understanding of the broader issue of gender differences in illness behaviour. From an applied point of view, an improved comprehension of the mechanisms behind the gender gap in sickness absence may be informative for policies and interventions aimed at safeguarding gender equality in the labour market and reducing sickness absence.

### Gender differences in sickness absence attitudes and norms

Attitudes towards sickness absence might differ between men and women because widely held gender stereotypes in society shape different expectations of when sickness absence is acceptable and when it is not [2]. For example, traditional female stereotypes of being weak and dependent [25,26] may legitimate sickness absence for women to a larger degree than for men, while traditional male role characteristics, such as competitiveness and independence [25,26], may make men less prone to accept sickness absence. Moreover, by virtue of their typical role as primary caregivers, women may be more motivated than men by the concern that a health problem threatens the fulfilment of caregiving duties. Such concerns may also make sickness absence more legitimate for women than men. A previous study suggests that controlled for gender, high levels of stereotypical male traits are related to reduced sickness absence risk, whereas stereotypical female traits tend to be associated with increased sickness absence risk [27]. The societal expectations and the practices of typical female role characteristics are also argued to be more health oriented than typical male characteristics [28]. Thus, there are several reasons to believe that there may be gender differences in sickness absence norms.

When considering research on attitudes towards work absence in general (without a specific focus on sickness-related absence), two previous studies suggest that women view absence from work as more legitimate than men do. The first study was based on survey data from 444 Canadian business school graduates [16], while the second study comprised cross-cultural survey data from 1,535 respondents distributed in nine nations [17]. The two studies used the same scale to assess the respondents’ general perception of absenteeism as a legitimate work behaviour with some of the items tapping into the view of absence as inevitable, understandable and punishable. Both studies found women to be more forgiving of work absence than men. Yet, when reasons for work absence were stated, women and men did not differ in work absence tolerance [17].

We identified two studies that examined social acceptability of sickness absence for women and men. Patton and Johns [2] analysed 167 articles on female absenteeism published in *The New York Times* over a 100-year period and concluded that gendered work absence norms do exist on a societal level. More specifically, the study indicated higher acceptance of sickness absence for women than for men based on general stereotypes related to women’s double workload of domestic duties and paid work, women’s frailer health and women’s lower work commitment. However, a second study by Patton [29] based on factorial survey data from 454 managers and professionals did not find differences in judgments of work absence due to illness based on absentee gender.

Only one previous study has examined gender differences in leniency towards sickness absence. By linking survey data from 226 health care workers to employer records on sickness absence, a Norwegian study found no significant differences between women and men in their attitudes towards sickness absence [30]. However, the study is limited by examining a rather specific group of employees in a female dominated profession (health care workers) and by employing a rather complex measure of attitudes that blends attitudes of shirking from work with attitudes towards more legitimate work absence due to sickness. Large scale studies using a representative sample and providing more detailed information about gender differences by using well-defined measures of attitudes towards sickness absence are therefore needed.

In conclusion, previous research on gender differences in sickness absence attitudes and norms is limited and the results are mixed. The few available studies indicate that women may view sickness-related work absence differently from men and that the social acceptance of sickness absence may differ by gender. Given the large gender gap, we expect more tolerant sickness absence attitudes among women than among men as well as higher social acceptance of women’s sick leave than men’s:

Hypothesis 1: Women have more tolerant attitudes towards sickness absence than men and thus judge sickness absence as reasonable more often than men do.
Hypothesis 2: Social norms of sickness absence favour women–that is, both men and women have more tolerant attitudes towards women being absent from work because of sickness than towards men being absent because of sickness.

### Differences in sickness absence norms by occupational gender composition

Several studies consider occupation to be an integrated component of gender stereotypes and suggest that occupational information evokes associations with gender roles and gender-stereotypical traits of the employee [31–34]. For example, employees in male-dominated occupations are considered to have stronger leadership skills, while employees in female-dominated occupations are viewed as more socially sensitive, regardless of employee gender [33]. People also seem to draw conclusions about a person’s occupation according to gender roles or gender-stereotypical trait information [32,35]. The judgment of an occupation as gender stereotyped is also repeatedly shown to reflect the statistical proportion of men and women in occupations [31,36]. Moreover, cross-national data from 41 countries confirm that the five most female-dominated occupations in the world–which include kindergarten teaching, nursing and secretarial work–typically involve socially sensitive and care-related tasks and are seldom characterised by leadership responsibilities [37].

In sum, the research literature implies that gender-dominated occupations are associated with gender roles and stereotypes. Accordingly, gendered occupations may prompt gender-stereotypical associations that influence the legitimisation of sickness absence. Given previous arguments about how female gender roles seem more compatible with sickness absence than male gender roles, we suggest that sickness absence acceptance may be greater for female-dominated occupations, which are typically associated with female gender roles.

So far, sickness absence norms in relation to gendered occupations have not been tested, but several studies suggest a tendency of higher sickness absence rates in female-dominated occupations or workplaces [38,39]. This tendency could imply that sickness absence norms are more lenient in cases of female-dominated occupations compared to male-dominated or gender-integrated occupations, particularly because past research indicates that female-dominated occupations are not unhealthier than male-dominated occupations are [12,13]. We posit the following hypothesis:

Hypothesis 3: Employees face more tolerant social norms of sickness absence in female-dominated than in male-dominated or gender-integrated occupations.

### The national context

Norway, adhering to the Nordic welfare model, is characterised by high participation of women in education and the workforce, as well as by shared housework and childcare [40,41]. However, despite Norway being a gender-equal welfare state, Norway’s labour market remains remarkably gender segregated and women have substantially higher sickness absence than men [13,41–43]. The gender difference in sickness absence is mainly evident for physician-certified sickness absence. In 2017, women had, on average, 72% higher physician-certified sickness absence than men, compared with 33% higher self-certified sickness absence than men [42,43]. The present study therefore concentrates on the evaluation of longer sickness absences that may qualify for physician-certification.

Norwegian employees may receive sickness absence compensation for up to one year. The employee’s own declaration (self-certification) that the absence is due to sickness is sufficient for the first few days (either three or eight in most firms); for longer absence periods, certification from a physician is required. The level of compensation is 100% up to a ceiling, and the public sector and many private sector firms offer full compensation even for higher earnings. The generous sick-pay scheme in Norway could provide more opportunities for non-financial factors to affect sickness absence than less favourable sick-pay schemes in other countries, making Norway an interesting case for studying gender differences in sickness absence attitudes and norms. Moreover, due to high levels of sickness absence, the costs of illegitimate absenteeism–that is, abuse of the generous sick-pay scheme–is more of an expressed concern in Norway than the costs of presenteeism–that is, employees going to work when sick, infecting colleagues and causing productivity loss.

## Methods

To examine whether or not men and women judge sickness absence differently, and whether or not men and women are judged differently when it comes to sickness absence, we conducted a factorial survey experiment in spring 2016, administered by the market research firm Kantar TNS.

### Procedure and participants

The study sample was drawn from a general-purpose, web-based panel established and managed by Kantar TNS. The Kantar panel consists of approximately 45,000 participants over the age of 15 who have been recruited to join the panel after participating in surveys conducted by the market research firm. Panel participants are usually invited to partake in one or two surveys a month. Participation in the panel is voluntary, but survey participation earns points that can be converted into selected gift items or gift vouchers, or donated to charity. Upon panel registration, participants provide background information about themselves to facilitate the selection process of participants for future surveys. In the present study, employment was a prerequisite for participation. Accordingly, 26,450 of the panel participants were eligible to partake in the survey.

The study questionnaire was sent by email to a random sample of 3,700 eligible panel participants (stratified by gender). In all, 59% of the invited participants opened the form (n = 2,176). Of these, 66 persons did not complete the form, while 310 persons met a “closed door” (i.e. all vignette alternatives were already answered when they opened the form). This recruitment approach ensured that exactly 1,800 respondents (900 women and 900 men) answered a form. The Data Protection Official for Research at The Norwegian Social Science Data Services approved the study. Moreover, the data file made available to the research group by Kantar TNS was without any kind of personal identifiers, and thus fully anonymous.

### The factorial survey approach

The factorial experimental method is particularly suitable for identifying individuals’ decision or evaluation principles [44]. The respondents are presented with descriptions of hypothetical scenarios (so-called vignettes), resembling real-life decision-making situations, and then asked to make a judgment. Across the vignettes, different factors are experimentally varied in order to estimate the impact of these multi-dimensional stimuli on the evaluation of the dependent variable.

In our survey, each vignette describes an employee, either male or female, in a specific occupation and with a specific health issue, and the respondents are asked to judge the reasonableness of sick leave in the situation. More precisely, the respondents are informed that the vignette-person has already been at home for three days of self-certified sickness absence but now thinks they need more time before returning to work. The respondents are then asked whether they think it is reasonable for the vignette-person to receive a physician-certified sick leave in the situation, with response categories “completely unreasonable” (1), “fairly unreasonable” (2), “fairly reasonable” (3), and “completely reasonable” (4), in addition to “don’t know” (see Appendix for the introductory text and a vignette example).

Our main dimension of interest is gender. In order to ensure that our findings in relation to gender differences (or lack thereof) in attitudes and/or social norms are not limited to a small number of scenarios, we included as many as 90 occupations and 30 diagnoses in the vignettes. To emphasise, we are not interested in the effects of a particular occupation or particular diagnosis, but in the effects of gender across a large number of situations. However, it is possible to combine the occupations and diagnoses into overall dimensions and test the effects of these–for example the importance of gender composition of an occupation. We selected occupations from the Norwegian State Register of Employers and Employees that represented different levels of female-dominated, male-dominated and gender-balanced occupations, as well as high-, middle- and low-status occupations [45]. For the diagnoses we used the Norwegian Labour and Welfare Administration’s statistics to choose examples among the most common diagnostic categories for sickness certification in Norway (i.e. mental illnesses, musculoskeletal disorders, headaches and dizziness, contagious respiratory illnesses and pregnancy complications). We also included some vignettes with examples of work- and family-related socio-psychological problems (i.e. work conflict, care responsibility for family members) instead of medical diagnoses (13% of the total number of vignettes). Vignette diagnoses concerning pregnancy complications were also included in the study design among female vignette-persons (7% of the total number of vignettes), because sickness absence tolerance due to such complications are planned to be examined as part of another publication. These vignettes were excluded from the present study because such vignettes could not be gender balanced.

To avoid the risk of fatigue, boredom or unwanted methodological effects such as response heuristics [44], we decided that each respondent would not have to judge more than six vignettes. With 90 occupations, 30 diagnoses and 2 genders, the total number of possible unique vignettes (the vignette universe) is 5,400 (90 x 30 x 2). Our data set includes all of these vignettes, divided into 900 questionnaires (5,400 / 6 = 900) in the following manner:

- The 2,700 exhaustive combinations of occupation and diagnosis were combined six and six into 450 questionnaires, in such a way that no questionnaire would contain the same diagnosis or the same occupation.
- Three of the vignettes in each questionnaire were randomly assigned female gender and three male gender (except where there was a pregnancy diagnosis included and the vignette person naturally had to be female).
- The order in which the six vignettes (and thus also specific diagnoses, occupations or genders) were presented within the individual questionnaire was random.
- For each of the 450 questionnaires we created a mirror image with reverse gender distribution for the six vignettes.

Each of the 900 unique questionnaire forms was answered by both a female and a male employee, giving us 1,800 respondents and 10,800 vignettes to analyse. The questionnaires were randomly assigned to respondents within the female and male sample. Since the sample of female and male respondents answered the exact same 900 forms, gender differences in sickness absence attitudes could not be influenced by order effects for the vignettes. Similarly, since each questionnaire had a mirror image with reverse gender distribution for the six vignettes, order effects cannot be the explanation for differences relating to gender of the vignette person (and gender differences in social norms). The data are fully available in S1 File.

### Statistical analysis

Our four-level dependent variable is most appropriately considered as an ordinal scale, and ordinal logistic regression would seem like a reasonable method. This model assumes, however, that the effect of the explanatory variable is identical irrespective of the cut point (e.g. whether it is set between categories one and two or between categories three and four; the so-called parallel regression or proportional odds assumption). The validity of this assumption can be evaluated by estimating three binary logistic regressions, one for each possible dichotomisation of the four-category variable, and then testing the null hypothesis that each of the coefficients are identical across the three regressions. As shown below, this hypothesis is rejected in the present case, and we therefore present the full set of binary logistic regressions. Since the respondent judges several vignettes, the measurements from each respondent have correlated error terms. Consequently, we employ robust standard errors that take clustering into account [46]. To ensure the experimental condition of the survey (i.e. an equal number of men and women featured in the vignettes), vignettes describing pregnancy-related diagnostic categories (n = 720) are excluded from all analyses.

## Results

### Descriptive statistics

The final sample consisted of 1,800 gainfully employed respondents, with 50% women (n = 900) and an average age of 47 years (*SD* = 14; range 18–83). In all, 48.8% of the respondents had college or university education, and 69.1% were living with a partner at the time of the interview. Furthermore, 58.8% of the women and 45.3% of the men reported to have had at least one sickness absence spell during the previous 12 months, yielding a 13.5 percentage-point gender gap in self-reported sickness absence.

The 10,080 vignettes constituted the analytical units in our analyses (sick leave judgments). Overall, respondents were quite accepting of sickness absence in the situations described; on average, 27.6% found sickness absence to be “perfectly reasonable”, 40.4% found it “fairly reasonable”, 20.8% found it “fairly unreasonable”, and only 7.0% answered “perfectly unreasonable”. Vignettes with the response “don’t know” constituted 4.2% (n = 428) of the vignettes and were excluded from the regression analyses.

Sick leave judgments varied considerably across vignette occupations; the percentage answering (“perfectly” or “fairly”) “reasonable” ranged from 50.0 to 84.8, and the percentage with (“perfectly” or “fairly”) “unreasonable” ratings varied from 13.4 to 46.4. Table 1 shows the ten occupations with highest “reasonable” ratings and the ten occupations with highest “unreasonable” ratings. The list of occupations with high acceptance of sickness absence included health-related work (nurse, hospital doctor) as well as other occupations where mistakes might have fatal consequences (truck driver, air traffic controller) and which involve potentially heavy manual work (sawmill production worker, firefighter). The list of occupations with low acceptance of sickness absence included typical office work, but also jobs with extensive customer contact (interpreter, bank customer service representative).

**Table 1:** The ten occupations where sickness absence was rated most frequently as “perfectly or fairly reasonable” and most frequently as “perfectly or fairly unreasonable”.

Sick leave judgments of vignette occupation			
Perfectly or fairly reasonable	%	Perfectly or fairly unreasonable	%
Sawmill production worker	84.8	Telephone salesperson	46.4
Assistant air traffic controller	80.4	Interpreter	38.1
Plumber	78.6	Accountant	36.9
Truck driver	78.4	Bank customer service representative	36.9
Auxiliary nurse	78.2	Professor	35.4
Nurse	77.7	Head librarian	35.1
Firefighter	75.9	Civil engineer in the oil industry	35.1
Kitchen help	75.9	Journalist	34.2
Hospital doctor	75.7	Gardener	34.2
Scaffold builder	75.5	Administrative officer	34.2

### Gender differences in sick leave judgments

**Fig 1:**
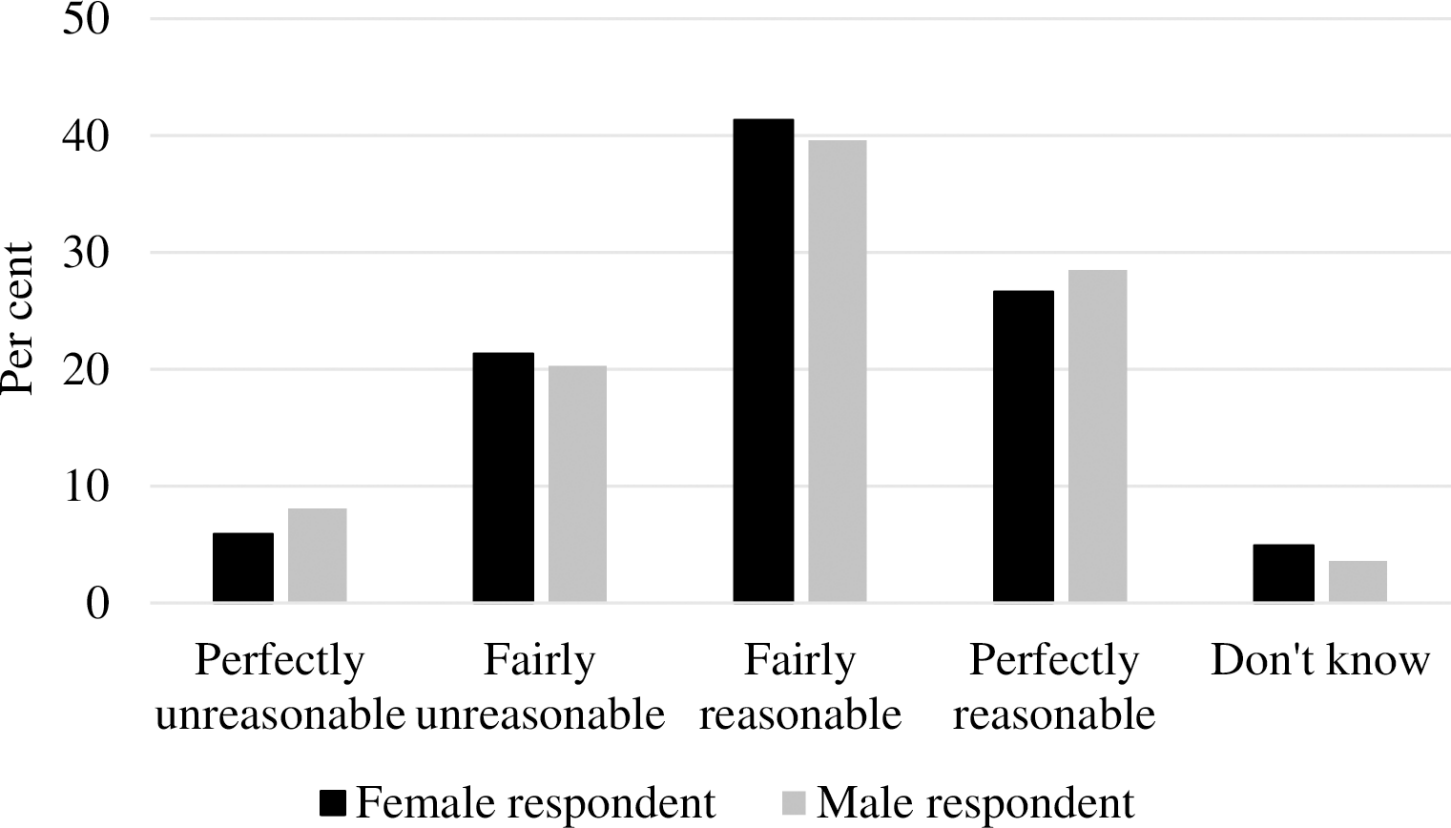
Distribution of sick leave judgments by respondent gender (%).

**Fig 2:**
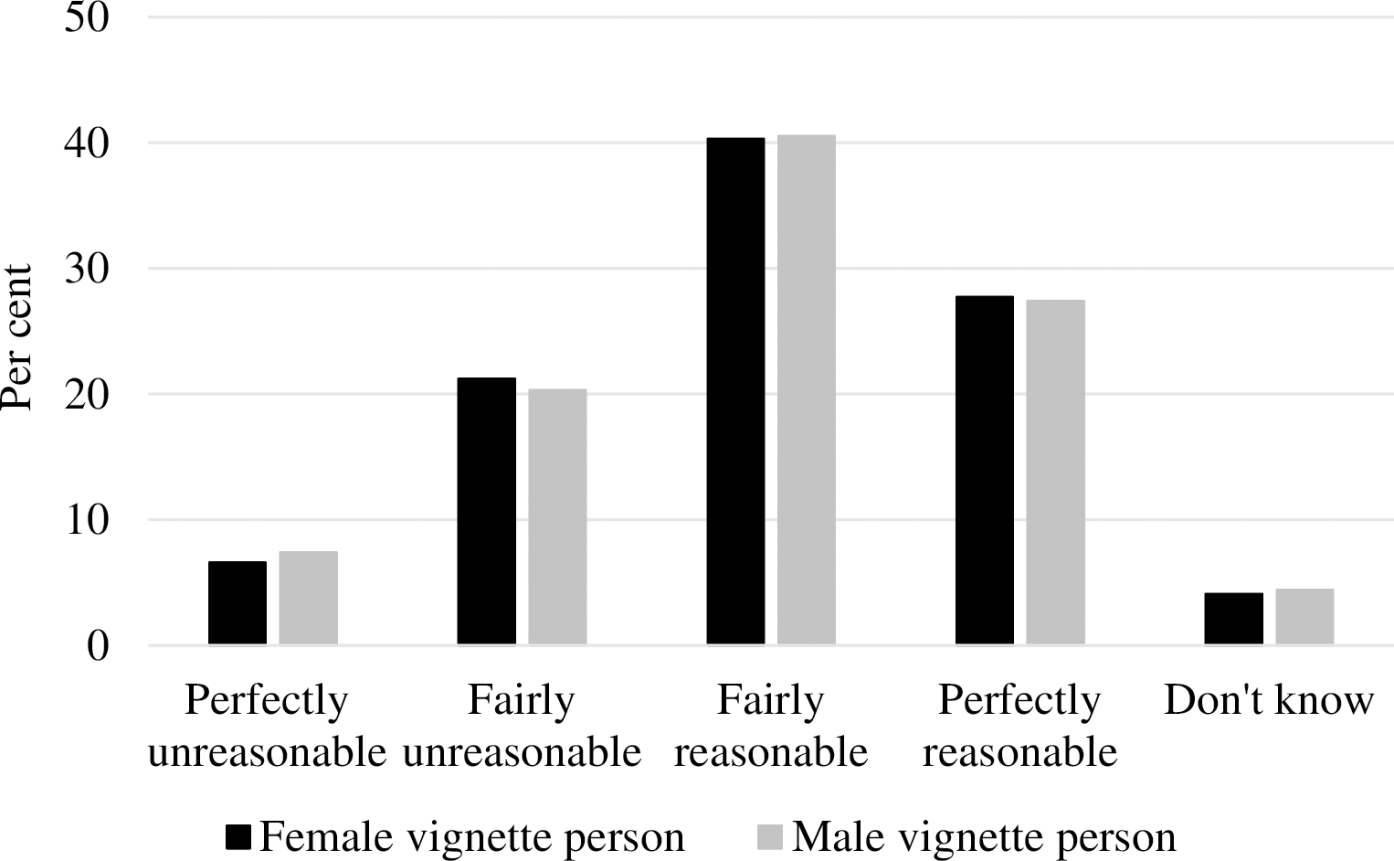
Distribution of sick leave judgments by vignette gender (%).

Turning to gender comparisons, Fig 1 shows the distribution of sick leave judgments by respondent gender. As displayed, men’s and women’s ratings were very similar, but there seemed to be a small tendency for men’s ratings to be more polarised than women’s, particularly regarding the “perfectly unreasonable” category. Women also came across as slightly more indecisive in their sick leave judgments than men were, illustrated by a 1.3 percentage-point gender difference in “don’t know” responses. Fig 2 presents the distribution of sick leave judgments by male and female vignette person. As shown, the respondents’ sick leave judgments were even more similar between male and female vignettes, indicating that sick leave judgments did not depend on vignette gender.

We tested hypotheses 1 and 2 by regressing sick leave judgments simultaneously on respondent gender and vignette gender. When conducting separate analyses for the three possible cut points on the vignette responses to test the proportional odds assumption of the ordinal logistic model (Table 2), this assumption was clearly rejected (χ^2^ = 18.56, df = 4, *p* = .001). In the following, we therefore present results from binary logistic regressions for each cut point.

**Table 2:** Logistic regression results with sick leave judgments regressed on respondent gender and vignette gender, with and without an interaction term. Separate analyses for alternative cut points on the dependent variable.

	Responses 2–4 vs. Response 1	Responses 3–4 vs. Responses 1–2	Response 4 vs. Responses 1–3
	OR (95% CI)	OR (95% CI)	OR (95% CI)
	**Model 1**		
Respondent gender	1.39** (1.14–1.70)	1.04 (0.93–1.17)	0.93 (0.81–1.07)
Vignette gender	1.13 (0.98–1.31)	1.00 (0.92–1.08)	1.01 (0.94–1.09)
Constant	10.27** (8.87–11.89)	2.40** (2.19–2.64)	0.42** (0.37–0.46)
	**Model 2**		
Respondent gender	1.40** (1.10–1.78)	1.08 (0.94–1.25)	0.96 (0.82–1.13)
Vignette gender	1.14 (0.94–1.38)	1.04 (0.93–1.15)	1.05 (0.95–1.16)
Resp. gender x Vign. gender	0.99 (0.74–1.32)	0.93 (0.79–1.09)	0.93 (0.80–1.09)
Constant	10.25** (8.75–11.99)	2.36** (2.14–2.60)	0.41** (0.37–0.46)

Response 1 = “perfectly unreasonable”; Response 2 = “fairly unreasonable”; Response 3 = “fairly reasonable”; Response 4 = “perfectly reasonable”. Vignettes with pregnancy-related diagnoses and “don’t know” responses are excluded. Number of vignettes: 9,652; number of respondents: 1,790. Gender is coded as male = 0 and female = 1.

* *p* < .05

** *p* < .01.

As shown in Table 2, only one cut-point analysis yielded a significant gender difference. Women had, compared to men, 39% higher odds of rating the vignettes as “fairly unreasonable”, “fairly reasonable” or “perfectly reasonable” than “perfectly unreasonable” (Responses 2–4 versus Response 1) than men (*p* < .01). This finding confirms the observation from Fig 1 suggesting that female respondents were less likely to use the “perfectly unreasonable” category, thereby displaying slightly more tolerant or less strict attitudes towards sickness absence than male respondents. However, this result is only partly supporting Hypothesis 1. When examining the effects of vignette gender, none of the results across all three cut points on the dependent variable revealed a significant difference in sick leave judgments according to vignette gender (*p* > .05). The results substantiate the similarities in judgments observed in Fig 2; thus, Hypothesis 2 was not supported. Adding an interaction term of the respondents’ gender and the vignettes’ gender (Model 2) did not reveal a gender difference in the likelihood of judging sickness absence differently depending on the vignette gender at any cut point (*p* > .05).

We also conducted additional age-stratified analyses to examine whether sick leave judgments varied across different age groups. For this purpose, we included two dummy variables in the regression equation to contrast the age groups 35–60 and 61–83 years, respectively, with the youngest participants (age 18–34 years). Moreover, we included interaction terms of both age group indicators with both respondent gender and vignette gender, and tested the null hypothesis that all coefficients for the interaction terms were jointly zero (i.e. that all gender coefficients were identical across age groups). This was done separately for each of the three cut-point specific regressions. The results showed that the null hypothesis could not be rejected (Responses 2–4 vs. Response 1: χ^2^ = 6.73, df = 4, *p* = 0.151; Responses 1–2 vs. Responses 3–4: χ^2^ = 0.88, df = 4, *p* = 0.928; Response 4 vs. Responses 1–3 χ^2^ = 2.95, df = 4, *p* = 0.566).

**Fig 3:**
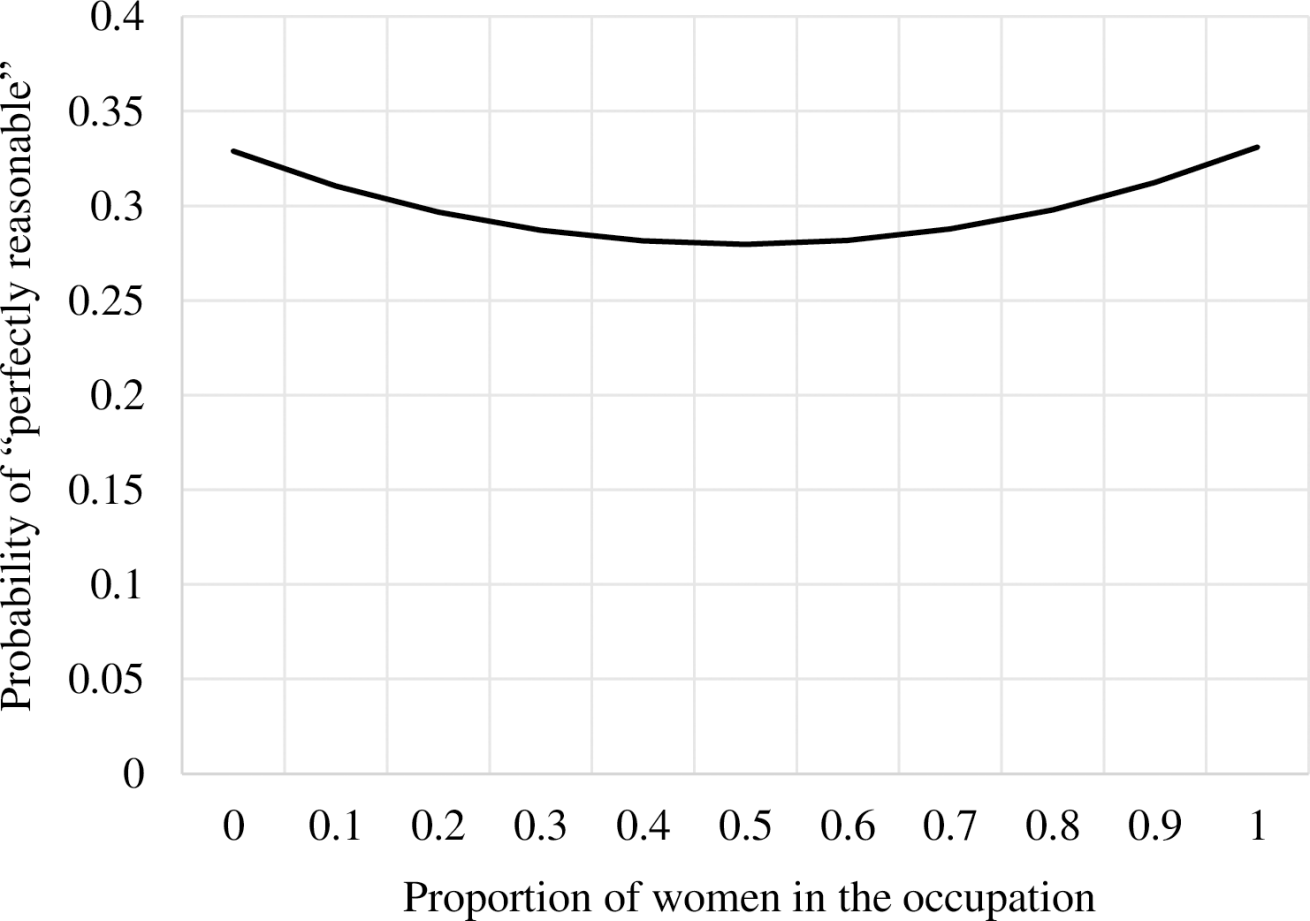
Probability of complete agreement (“perfectly reasonable”) that sick leave is reasonable as a function of the proportion of women in the occupation. Controlled for respondent gender and vignette gender. Numbers based on the analysis results from cut off “Response 4 versus Responses 1–3”.

Hypothesis 3 was tested by conducting binary logistic regression analyses of sick leave judgments on the proportion of women in the vignette occupation, with control for respondent gender and vignette gender. As shown in Table 3, all three separate analyses for alternative cut points on sick leave judgments showed a negative relationship between proportion of women in the vignette occupation and favourable judgments. However, to consider non-linearity, a squared term of the proportion of women in the vignette occupation was also included in the analyses. The results suggest a U-shaped relationship between more favourable sick leave judgments and the proportion of women in the vignette occupations for all three cut-point analyses. Fig 3 illustrates this finding by the plotting of probabilities for one of the cut points: “perfectly reasonable” as a function of the proportion of women in the occupation. As shown, both male-dominated and female-dominated occupations evoked a higher likelihood for lenient sick leave judgments than gender-integrated occupations, irrespective of vignette gender. The plot also suggests that employees in fully gender-integrated occupations are judged in the least lenient manner and employees in fully gender-dominated occupations are judged in the most lenient manner. Hence, these findings only partially support Hypothesis 3, because employees in both male- and female-dominated occupations seem to be judged in a similarly favourable manner compared to employees in gender-integrated occupations. Finally, we rerun all analyses without including the 1,440 vignettes that did not strictly concern medical diagnoses (i.e. work- and family-related socio-psychological problems), but these analyses did not change the study results considerably.

**Table 3:** Logistic regression results with sick leave judgments regressed on respondent gender, vignette gender and proportion of women in the vignette occupation. Separate analyses for alternative cut points on the dependent variable.

	Responses 2–4 vs. Response 1	Responses 3–4 vs. Responses 1–2	Response 4 vs. Responses 1–3
	OR (95% CI)	OR (95% CI)	OR (95% CI)
Respondent gender	1.39** (1.14–1.70)	1.04 (0.93–1.17)	0.93 (0.81–1.06)
Vignette gender	1.14 **(**0.98–1.31)	1.00 (0.92–1.08)	1.01 (0.94–1.09)
Prop. women	0.35* (0.13–0.92)	0.25** (0.14–0.43)	0.39** (0.23–0.65)
Prop. women squared	2.57* (1.01–6.52)	3.53** (2.07–6.01)	2.59** (1.57–4.25)
Constant	12.60** (9.81–16.17)	3.16** (2.73–3.66)	0.49** (0.42–0.56)

Response 1 = “perfectly unreasonable”; Response 2 = “fairly unreasonable”; Response 3 = “fairly reasonable”; Response 4 = “perfectly reasonable”. Vignettes with pregnancy-related diagnoses and “don’t know” responses are excluded. Number of vignettes: 9,652; number of respondents: 1,790. Gender is coded as male = 0 and female = 1.

* *p* < .05

** *p* < .01.

## Discussion

The main purpose of this study was to examine potential gender differences in attitudes and norms of sickness absence. Altogether, the analyses did not support such differences. Overall, women and men judged sickness absence similarly, even though one of the analyses suggested that women consider sickness absence as “perfectly unreasonable” less frequently than men. Furthermore, we did not find evidence of sickness absence norms favouring women–that is, men and women were not judged differently when absent because of sickness. However, the occupational gender composition was associated with the respondents’ sick leave judgments, suggesting that, regardless of gender, employees in both male- and female-dominated occupations faced more tolerant norms of sickness absence than employees in gender-integrated occupations.

### Strengths and limitations

Since few gender differences were found in the present study, we must discuss whether limitations of the study design could have contributed to the lack of association. One limitation is that the study sample comprises individuals who are willing to participate in surveys on a regular basis and thus may not be representative of the general Norwegian population. Nevertheless, there is no obvious reason why people who frequently participate in surveys, or who in other ways do not perfectly reflect the average Norwegian, should have either stronger or weaker gender-biased attitudes concerning sickness absence legitimacy.

Another limitation is that attitudes (and norms) are hypothetical constructs that are difficult to measure [47]. Although the elaborated situational descriptions in survey vignettes improves the possibilities of stimuli standardisation (i.e. less abstract, vague and indirect questioning) and reduces the likelihood of responses being influenced by social desirability bias compared to traditional survey questions [44,48], it is not a given that respondents’ judgments are generalisable to real life. On the one hand, the scenarios could have been too specific, thereby restricting the influence of gender norms on sick leave judgments. For example, with scenarios that only indicate a diagnosis (i.e. that lack symptom description), there might be more leeway for judgments to be influenced by gender differences in health focus and the challenges that a health problem may cause. On the other hand, one might also argue that the scenarios were not specific enough–that simply describing sick leave scenarios is not sufficiently specific to reflect the actual norms that individuals face in real-life situations, potentially weakening the effect of societal sickness-absence expectations on respondents’ judgments. Still, our careful efforts to create sick leave scenarios that represent the most common diagnostic categories for sickness certification, a wide range of occupations and our experimental condition should strengthen the credibility of the scenarios and the generalisability of judgments. In this respect, the data set is also uniquely comprehensive and innovative compared to previous studies in the field. We also acknowledge the possibility of complex interplays between personal characteristics not assessed in this study and vignette characteristics. For example, the relationship between vignette occupation and sick leave judgments may vary according to respondents’ own occupation. However, respondents’ occupation was not assessed in the present study.

A further limitation is that the analyses are restricted to the Norwegian labour market. This is not an obvious explanation for our findings, however, since gender differences in sickness absence are greater in Norway than in most other countries. Nevertheless, only future research can provide information on whether our findings are generalisable to other samples and countries with different sick leave policies and labour market characteristics.

### Equally tolerant sickness absence attitudes among women and men

Our first hypothesis predicting that women judge sickness absence as reasonable more often than men was not supported overall. Although one of the analyses suggests that women are slightly less likely to exclude completely the legitimacy of sickness absence in some instances, we cannot conclude that women generally have more tolerant attitudes than men. Therefore, our results imply that women and men actually judge sickness absence similarly. The results are partly in disagreement with those of two previous studies that used the same measure of work absence legitimacy and showed that women generally had a broader tolerance of absence from work than men [16,17]. However, the measure applied in these two studies did not include attitudes towards different reasons for work absence. Nonetheless, when Addae and colleagues [17] additionally measured views of absence legitimacy using work absence scenarios that also stated reason for work absence, men and women, in line with our results, did not differ in work absence tolerance. Still, illness was not included as a reason for work absence in their scenarios. The present study is therefore the first to measure gender differences in sickness absence attitudes using sickness absence scenarios and a comprehensive population-based sample. Thus, the present study provides solid support for the notion that gender differences in sickness absence attitudes are small and may therefore be of minor importance in explaining the gender gap in sickness absence.

### Women and men face similar sickness absence norms

Our second hypothesis postulated that people have more tolerant attitudes to women’s sickness absence than to men’s. As no difference in the evaluation of men’s and women’s sickness absence was found, this hypothesis was not supported either. The results correspond to those of Patton [29], which also found no differences in judgments of work absence based on absentee gender in an American study sample. However, the present study results seem to diverge from those of another American study that examined gendered work absence norms. From their analysis of newspaper content, Patton and Johns [2] concluded that work absence norms are legitimising work absence for women because of common stereotypes such as women’s weaker health and greater loads of domestic and paid work compared to men. The different result may reflect temporal differences as Patton and Johns’ analyses covered a long historical period and only six observations (newspaper articles) were post-year 2000. In addition, the methodological differences are substantial because, while we measured attitudes and norms as they may affect the behaviour of specific individuals in concrete situations, Patton and Johns dealt with more general ideas and attitudes found in the public discourse.

### Favourable sickness absence norms for gender-dominated occupations

The third hypothesis, predicting that employees face more tolerant norms of sickness absence in female-dominated occupations than in male-dominated or gender-integrated occupations was partly supported in the present study. Our findings are consistent with the idea that sickness absence norms are “gendered”, but only if this means that sickness absence norms are more lenient in both female- and male-dominated occupations than in gender-integrated occupations. The similarity in judgments between male- and female-dominated occupations, irrespective of employee gender, implies that we cannot conclude that favourable sickness absence norms for gender-dominated occupations are influenced by gender stereotypes or their gender balance per se.

The U-shaped association between sick leave judgments and occupational gender composition corresponds with studies showing that sickness absence rates are higher in both strongly male- and strongly female-dominated occupations than in gender-integrated occupations [1,49]. Sickness absence rates also seem to decrease with higher job level (i.e. level of autonomy and authority in the job) for both men and women in gender-dominated occupations, while this pattern is less obvious in gender-integrated occupations [50]. Higher sickness absence rates in strongly gender-dominated occupations may partly reflect their generally greater incompatibility with performing work tasks while having a health issue compared to gender-integrated occupations. Likewise, more lenient sick leave judgments for highly gender-dominated occupations in the present study could be the result of the type of job tasks that respondents associate with these occupations. In other words, the typically heavier manual work and less autonomy and flexibility of these occupations might be judged as more compatible with sickness absenteeism and less compatible with sickness presenteeism than more gender-integrated occupations such as office or managerial positions.

### General discussion

In view of the substantial gender gap in sickness absence and the common notion that women typically deal with double workloads of domestic and paid work, it is surprising that sickness absence norms do not seem to favour women at all. As noted above, there is also a widespread assumption in broader research on illness behaviour that gender differences in such behaviours are to a considerable extent an outcome of gendered attitudes and norms [28,51]. Nevertheless, not all research on illness behaviour supports this idea. For instance, Hunt and colleagues [52] found that among those known to have either headache or back pain symptoms, only small if any gender difference in consultations was found. One interpretation of this finding is that men and women differ primarily in their propensity to define, or not to define, something as a health problem; if a condition is defined as a health issue, the norms and attitudes may be similar for men and women.

A further possibility is that norms and attitudes have changed over time. Although gender stereotypes might generally not have kept up with the rapid increase of women in the workforce in recent decades, the increasing gender equality in workforce participation may have contributed to men and women having similar sickness absence attitudes today. Additionally, studies suggest that women overall do not have a lower commitment to work or lower work ethic than men [53,54], which may also explain the lack of gendered sickness absence attitudes in the present study. Moreover, the marked focus on the gender gap in the Norwegian public discourse over the last two decades might have altered sickness absence norms, resulting in lower tolerance for female sickness absence in later years, thereby cancelling any prior gender difference in such norms.

Future studies may profit from exploring whether gendered attitudes and norms of sickness absence exist in crucial groups. For example, stricter guidelines for physicians certifying sick leave are related to reduced sickness absence [19]; thus, general practitioners have a participatory role in the sickness absence rate and could possibly contribute to the gender gap in sickness absence. Also, factorial surveys examining sickness absence attitudes in other samples and countries are needed to establish the generalisability of the study results.

The limited understanding of the gender gap could be problematic. The higher sickness absenteeism among women may result in gender discrimination in the workplace and in employers’ hiring practices, since such absence is often associated with increased costs and work disruption [55]. Sickness absence is also linked to reduced income and career opportunities and to disability and unemployment for the individual [56,57]. We consider the lack of gendered attitudes and norms of sickness absence found in the present study to be an important contribution to the field. Notably, our study does not support the popular belief that women have higher sickness absence than men because of commonly gendered attitudes and norms in society. Hence, the study results do not indicate that low work engagement and work morale among women explain the gender gap in sickness absence.

## Conclusions

Insufficient explanations for the gender gap in sickness absence has raised speculation that gendered attitudes or norms promote female sickness absence. The higher sickness absence among women than among men, and speculation as to what is causing this gender gap, could harm gender equality in the labour market. It is therefore in the interests of society to explain the mechanisms underlying the gender difference in sickness absence. Moreover, knowledge about factors that may cause sickness absence might prove useful for reducing sickness absence rates for both men and women. The present study results suggest that societal attitudes and norms of sickness absence are unlikely to be important factors driving the gender gap. Accordingly, the results are informative for policies and interventions aimed at reducing the gender gap in sickness absence, since poor work morale or work engagement do not seem to shed light on the gender gap. Future research may benefit from examining whether similar results will be obtained in other countries with varying levels of gender equality in the labour force. Moreover, research on whether gendered norms of sickness absence exist in important groups of societal interest, such as among physicians who certify sick leave, may provide a better understanding of potential sources of gender differences in sickness absence.

## Appendix

### Introductory text for the vignettes

The respondents were met with the following introductory text before being presented the six vignettes:

In this survey, we want to know what you think is a reasonable cause for sick leave. We describe six different situations, in which a person has been home for three days of self-certified sick leave, but where the person thinks he/she needs more time before he/she returns to work. We ask you to evaluate, for each situation, whether you think it is *reasonable that the person receives a physician-certified sick leave in this situation*.

### Vignette example

A full vignette example is displayed below:

Frank works as a scaffold builder. He is afflicted by a stiff and painful neck and pain in both shoulders. The pain is not very strong, but present as a more or less constant ache. He notices a tendency of improvement when he can take it easy, while the pain is aggravated by stress. Frank has been at home for three days of self-certified sickness absence, but thinks that he needs more time before he returns to work. How reasonable or unreasonable do you think it is that Frank receives a physician-certified sick leave in this situation?

Each vignette was rated by four graded response categories; “perfectly unreasonable” (1), “fairly unreasonable” (2), “fairly reasonable” (3), and “perfectly reasonable” (4), in addition to “don’t know” (5).

## Supporting information

S1 File Full vignette dataset.xlsx. The file contains an Excel sheet with data tabulated under the tabs: “Data on the vignette level” and “Variable names and labels”. All 10,800 vignettes are included in this file.
(XLSX)
